# Identification, variation and transcription of pneumococcal repeat sequences

**DOI:** 10.1186/1471-2164-12-120

**Published:** 2011-02-18

**Authors:** Nicholas J Croucher, Georgios S Vernikos, Julian Parkhill, Stephen D Bentley

**Affiliations:** 1 Pathogen Genomics, The Wellcome Trust Sanger Institute, Wellcome Trust Genome Campus, Hinxton, Cambridge, CB10 1SA, UK

## Abstract

**Background:**

Small interspersed repeats are commonly found in many bacterial chromosomes. Two families of repeats (BOX and RUP) have previously been identified in the genome of *Streptococcus pneumoniae*, a nasopharyngeal commensal and respiratory pathogen of humans. However, little is known about the role they play in pneumococcal genetics.

**Results:**

Analysis of the genome of *S. pneumoniae *ATCC 700669 revealed the presence of a third repeat family, which we have named SPRITE. All three repeats are present at a reduced density in the genome of the closely related species *S. mitis*. However, they are almost entirely absent from all other streptococci, although a set of elements related to the pneumococcal BOX repeat was identified in the zoonotic pathogen *S. suis*. In conjunction with information regarding their distribution within the pneumococcal chromosome, this suggests that it is unlikely that these repeats are specialised sequences performing a particular role for the host, but rather that they constitute parasitic elements. However, comparing insertion sites between pneumococcal sequences indicates that they appear to transpose at a much lower rate than IS elements. Some large BOX elements in *S. pneumoniae *were found to encode open reading frames on both strands of the genome, whilst another was found to form a composite RNA structure with two T box riboswitches. In multiple cases, such BOX elements were demonstrated as being expressed using directional RNA-seq and RT-PCR.

**Conclusions:**

BOX, RUP and SPRITE repeats appear to have proliferated extensively throughout the pneumococcal chromosome during the species' past, but novel insertions are currently occurring at a relatively slow rate. Through their extensive secondary structures, they seem likely to affect the expression of genes with which they are co-transcribed. Software for annotation of these repeats is freely available from ftp://ftp.sanger.ac.uk/pub/pathogens/strep_repeats/.

## Background

Small interspersed repeats, spatially separated genomic regions of similar sequence typically < 200 bp in length, are frequently found in bacterial chromosomes [1]. These can be classified as either 'simple', when consisting of a single repeated unit, or 'composite', when comprised of a combination of different subsequences arranged in particular patterns [2]. For example, a number of enterobacterial species harbour many instances of the simple 127 bp Enterobacterial Repetitive Intergenic Consensus (ERIC) sequence [3] and hundreds of composite Bacterial Interspersed Mosaic Elements (BIMEs), which include multiple copies of the Palindromic Unit in a regular configuration. Similarly, *Neisseria meningitidis *genomes host simple 183 bp AT-rich Repeats and two families of more common, composite elements: 70-200 bp Neisserial Intergenic Mosaic Elements (NIMEs) and Correia Elements (CE), comprised of internal sequences up to 156 bp long delimited by 26 bp inverted repeats [4].

Many such repeat families are likely to be non-autonomous mobile parasitic elements, termed Miniature Inverted-repeat Transposable Elements (MITEs). These are characterized as being AT-rich, possessing terminal inverted repeats (TIR), having highly base-paired secondary structures and generating target site duplications (TSDs) on insertion [1]. In a number of cases, it has been proposed that repeats are mobilized by the transposases encoded by IS elements within the same host, based on similarities between the TIR of the MITE and the IS sequence. For instance, the *Nezha *MITE found in cyanobacteria is proposed to be mobilized by IS*Npu3*-like elements [5].

The tightly folded secondary structure characteristic of putative MITEs means they can impact on gene expression when they insert into transcribed regions. Some BIMEs, when inserted into operons, have been found to decrease the expression of downstream CDSs through acting as transcriptional attenuators [6]. By contrast, regions upstream of ERIC elements integrated into operons may be destabilised by the presence of the repeat when in a specific orientation, as it appears to trigger transcript cleavage through introducing a putative RNase E target site [7]. Similarly, there is evidence that CE act as a target site for RNase III-mediated endoribonucleolytic cleavage when transcribed [8,9]. CE insertions have also been found to influence gene expression through generating functional promoters in *N. meningitidis *[10]. As well as affecting transcriptional regulation, repeat sequences can alter the sequences of genes without disrupting their function. For instance, in *Rickettsia*, repeat element insertions have been found in both coding and non-coding genes that appear still to be functional [11,12].

*Streptococcus pneumoniae *is a nasopharyngeal commensal and major respiratory pathogen estimated to have caused almost 15 million cases of disease in 2000 [13]. The genome, typically around 2 Mb in size, is known to contain two types of small interspersed repeat. The first to be discovered was the BOX element, a composite repeat consisting of boxA and boxC sequences usually separated by a variable number of boxB elements arranged in a tandem array [14]. The variation in different strains' complements of these repeats has allowed them to form the basis of a PCR-based epidemiological typing scheme [15]. An early hypothesised function of BOX elements, based on their proximity to a number of genes involved in competence and pathogenesis, was that they might act as regulatory motifs [14], and subsequent experiments have shown that boxA and boxC elements are able to stimulate the expression of downstream genes, although boxB elements can have an opposing inhibitory effect, depending on their orientation [16]. A BOX element has also been hypothesised to increase the frequency of pneumococcal phase variation through affecting the regulation of neighbouring genes [17]. Similarity between the TIR of BOX elements and IS*Spn2*, a transposon found in *S. pneumoniae*, has been proposed as the basis for mobilization of these elements. Likewise a second repeat also present in high copy number in the pneumococcal genome, the simple 107 bp long Repeat Unit of Pneumococcus (RUP), has TIR similar to those of IS630-*Spn*1, another transposon commonly found in *S. pneumoniae *[18]. RUP were proposed to preferentially insert into or near IS elements, based on their distribution in a draft of the *S. pneumoniae *TIGR4 genome [19], leading to the suggestion that these elements may serve to limit the number of functional transposase genes in the chromosome [1].

Here we present an analysis of the distribution of pneumococcal small interspersed repeats throughout the publicly available streptococcal genomes and outline how these elements may have impacted upon the evolution of pneumococcal coding and non-coding genes.

## Results

### Three Families of Repeats are Present in the Pneumococcal Chromosome

**Figure 1:**
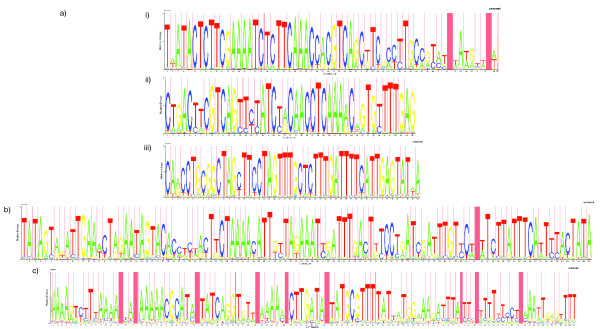
**HMM logos representing pneumococcal interspersed repeat sequences**. These images describe the HMMs used for sequence searches. Each column corresponds to a nucleotide in the repeat element. The total height of bases in each column represents how informative that position is in describing the element; the relative heights of the different bases indicate their respective emission probabilities in the model. Red shaded columns show positions where base insertions occur: the total width of these columns represents the expected number of inserted bases, whilst the dark shaded component indicates the probability that an insertion occurs. The repeats displayed are a) i) boxA, ii) boxB, iii) boxC, b) RUP and c) SPRITE.

**Figure 2:**
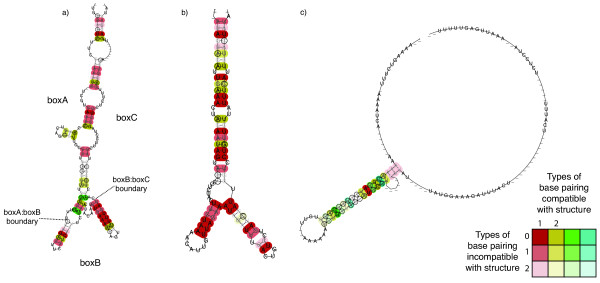
**Predicted repeat sequence secondary structures**. These images represent the predicted secondary structures of transcribed forms of the repeat sequence families: a) BOX, b) RUP and c) SPRITE. The structures were generated from an alignment of 30 sequences from the *S. pneumoniae *ATCC 700669 genome in each case; only BOX elements with a canonical A_1_B_1_C_1 _structure were used to produce the structure in a). The boundaries between the different subsequences are marked on the image. Base pairings are coloured according to their conservation in the alignment: the bolder the colour, the more strongly conserved the pairing of the bases, with different colours indicating different numbers of compatible interactions at equivalent sites in the structure (see key).

The curated output of RepeatScout revealed the presence of three distinct repeat families in the genome of *S. pneumoniae *ATCC 700669 [20]. One of these corresponded exactly to the ~107 bp RUP element. Another represented the reverse complement of the 3' end of BOX elements; consequently, to fully define such repeats, independent models for each of the BOX modules were then constructed. The third is a novel repeat element, which we shall refer to as the *Streptococcus pneumoniae *Rho-Independent Terminator-like Element (SPRITE), on the basis of its sequence and predicted secondary structure (Figures 1c and 2c).

Following refinement of the models (see Methods), the final HMMs used to identify the repeats are represented as logos in Figure 1. Overall, 125 BOX (composed of 422 modules), 110 RUP and 30 SPRITE elements were found in the ATCC 700669 genome; in addition, 17 lone box modules were found. All of the original examples used to define BOX and RUP elements were identified by this approach [14,18]. It seems likely that the lower frequency of the SPRITE repeat is the explanation as to why it was not characterised prior to the availability of complete genome sequences.

Each of the three families of repeats share at least some features of MITEs. All are typically < 200 bp in length; unsurprisingly, the modular BOX elements are the most variable in size, ranging from 67 bp to 637 bp. Both RUP and SPRITE are AT-rich relative to the *S. pneumoniae *genome (GC content of 39.5%), with mean GC levels of 27.5% and 28.1% respectively. Both BOX and RUP have been previously shown to have TIR and cause TSDs on insertion [14,16,18]. SPRITE repeats have comparatively shorter and simpler TIR (the tetranucleotide AAAA and the complement TTTT; Figure 1c). Any TSD produced by SPRITE insertions could not be established from the current dataset, because no instances of the repeat with an easily comparable empty site could be found in the available collection of sequences, and no clear evidence could be identified by examining the regions flanking insertions.

All three elements are predicted to form stem-loop structures if transcribed into an RNA form (Figure 2). The structure of BOX elements was generated from those elements with a canonical A_1_B_1_C_1 _sequence; notably, the folding of the boxB element is predicted to involve few interactions with the boxA and C elements that form the rest of the structure. If this folded RNA is functional, this characteristic may be permissive in allowing boxB to be absent, or present in multiple copies, without causing much disruption to the overall form of the transcript.

The SPRITE structure is less tightly folded than that of BOX or RUP, and consists of an 18 bp duplex followed by a relatively uridine-rich (~48% uridine) tract, seeming likely to imbue it with the properties of a Rho-independent terminator. However, the repeat's structure is distinctive in that both the stem duplex and T-rich tract are much longer than the ~10 bp size of both these features in typical streptococcal Rho-independent terminators [21]. Hence it appears that SPRITE are distinct from normal Firmicute terminators, although they may be able to function in such a capacity.

### Genomic Distribution of Pneumococcal Repeats

**Figure 3:**
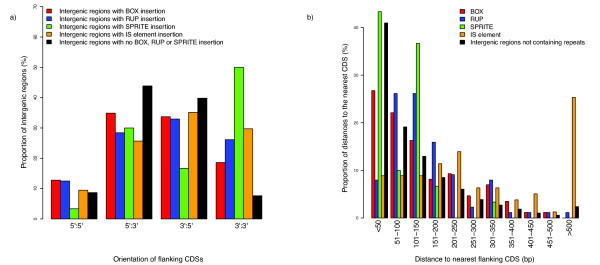
**Distribution of repeat sequences within the *S. pneumoniae *ATCC 700669 genome**. a) The orientation of the CDSs flanking different repeat elements. Data are shown for BOX, RUP, SPRITE and IS elements annotated in the chromosome; the black bars indicate the orientations of all neighbouring CDSs in the genome not separated by intervening small interspersed repeats. b) A comparison of the distance from repeat sequences, including IS elements, to the nearest CDS, and the distribution of lengths of intergenic sequences not containing repeats.

The distribution of these repeats relative to the protein coding genes of *S. pneumoniae *ATCC 700669 was examined. BOX, RUP and SPRITE were all found to mirror the coding bias of the sequence, with 60.8%, 60.9% and 63.3% of insertions on the leading strand of the genome, respectively. Although BOX elements have been found to affect gene regulation [16], they are only slightly overrepresented between divergently transcribed genes, and like RUP, SPRITE and IS elements, they are significantly overrepresented between convergently transcribed genes (Figure 3a; Table 1). This may be seen as evidence that these elements are mobile, parasitic entities: the regions downstream of CDS are less likely to be under strong selection pressures, and hence more likely to tolerate repeat element insertions, than upstream regulatory regions or intergenic sequences between cotranscribed genes. Most strongly enriched in these regions are SPRITE, which, given their resemblance to terminator sequences, seem the most probable to disrupt transcription if inserted upstream or between genes.

**Table 1:** Overrepresentation of repeats between convergently transcribed genes

Feature	No. Upstream of ≥ 1 CDS	No. Between Convergently Transcribed CDSs	P Value
**BOX**	70	16	0.0017
**RUP**	65	23	3.4 × 10^-7^
**SPRITE**	15	15	1.7 × 10^-9^
**IS element**	52	22	5.1 × 10^-8^
**Intergenic sequence**	1800	149	-

This table shows the results of testing for overrepresentation of repeat sequences in intergenic regions between convergently transcribed CDSs. For each repeat type, the number of insertions in the two different contexts were tested against the number of intergenic sites containing no short interspersed repeats in the same contexts (bottom row). The displayed P values were calculated from these 2 × 2 contingency tables using a two-tailed Fisher exact test.

Across the pneumococcal chromosome, the size of intergenic distances follows a gradually decaying distribution (Figure 3b). A similar pattern is observed with the distances between BOX elements and the nearest gene, whereas the density of RUP elements is greatest 50-150 bp from the nearest gene. IS elements have an even more pronounced tendency to be distant from neighbouring CDSs; this may reflect the greater potential disruption to gene expression caused by these longer repeats should they insert within, or near, functional transcripts. SPRITE sequences tend to be close to adjacent CDSs, with only one SPRITE found >200 bp from the nearest gene. This enrichment of SPRITE close to the 3' termini of CDS suggests they may have been co-opted by the pneumococcus into acting as functional transcriptional terminators.

**Figure 4:**
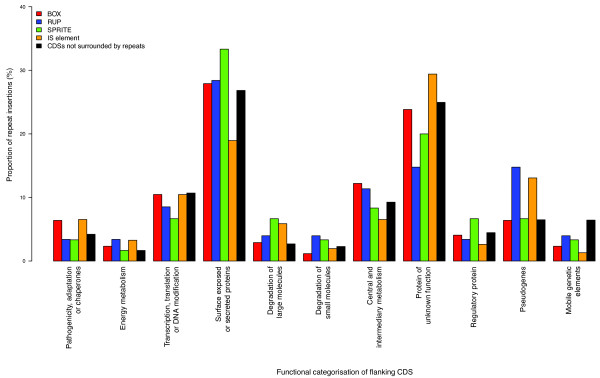
**Distribution of repeat sequences relative to CDS function**. Functional classification of CDSs adjacent to repeat sequences in the genome of *S. pneumoniae *ATCC 700669. For each type of repeat, both flanking CDSs were considered, and the proportions indicated by the graph. The black bars record the equivalent classification of CDSs with at least one associated intergenic region not containing a repeat sequence.

Few clear relationships can be ascertained by looking at the association between repeats and the functional classes of their flanking CDS (Figure 4). This again argues against a general role for these repeats as upstream regulatory elements coordinating transcriptional responses to stimuli, as has been previously suggested [14], because no informative overrepresentation of a repeat near CDSs with a particular function is observed. Furthermore, in agreement with Tettelin *et al *[19], no support for the hypothesised association between IS elements and RUP insertions can be found [18]. The positioning of repeat arrays next to genes encoding surface-exposed proteins that may trigger a host response, proposed as a mechanism for promoting horizontal transfer of CDS for antigenic proteins in *N. meningitidis *[22], is also not observed in *S. pneumoniae*. One apparent association, the preponderance of RUP elements and IS elements adjacent to pseudogenes, seems likely to reflect the tolerance of repeat insertions into regions of the genome that are no longer functional.

**Figure 5:**
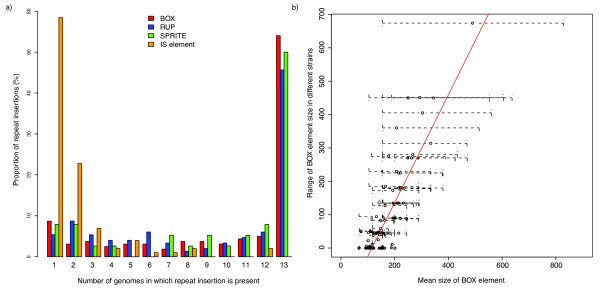
**Distribution of repeat sequences between pneumococcal genomes**. a) For each of the three families of small, interspersed repeat, and for IS elements, orthologous insertion events were defined between chromosomes (see Methods) and scored for presence or absence in the 13 complete pneumococcal clinical isolate chromosomes. The bar chart shows the proportion of insertions of each repeat element shared by a given number of pneumococcal sequences, ranging from insertions present in a single chromosome to those conserved among all strains. b) Variation in the length of BOX elements. For each BOX element identified in the chromosome sequences, the mean, minimum and maximum sizes of orthologous inserts in different strains was calculated. The graph shows the mean length plotted against the range in size, with the dotted bars showing the span of sizes detected.

The level of variation in repeat insertions between all publicly available complete *S. pneumoniae *genomes was also studied (Figure 5a). For all three small interspersed repeats, approximately half of the insertions are 'core', i.e. present in all sequenced strains. This contrasts with the distribution of autonomously mobile IS elements, of which the majority of insertions are present only in a single strain. This is likely to reflect IS elements having a comparatively higher transposition rate, while also being removed more quickly by selection. Assuming that the frequency of IS elements in the pneumococcal population is relatively stable over time, this implies that they are much more mobile than the small interspersed repeats. Despite the hypothesized transposition of RUP in *trans *by IS630-*Spn*1 elements, there is no clear evidence from this distribution between genomes that it is more mobile than BOX, which has a lower level of similarity to the TIR of IS*Spn2 *[16], or SPRITE, for which no significant similarity with pneumococcal IS TIR could be found.

One way in which BOX elements are observed to vary quite considerably is in their size (Figure 5b). Several mechanisms have been proposed to explain the fluctuation in the length of tandem repeat arrays, including slipped strand mispairing, unequal crossover during homologous recombination and circular excision followed by reinsertion [23]. Plotting the mean size of each BOX element insertion against the range of the lengths of the insertion in different genomes reveals a positive linear correlation (*R^2 ^*= 0.74, *p *< 2.2 × 10^-16^). This implies that the greater the average number of boxB repeats in a BOX element, the more likely that element is to vary by losing or acquiring these modules. Notably, all BOX elements with a large mean size exhibit considerable variation in length between strains. This result indicates that at the disparate loci at which BOX elements are found, there is significant variation in the rate of mechanisms that change the number of boxB modules in these arrays, or greatly differing levels of selection pressure constraining the size of these composite repeats.

### Repeat sequences in other streptococci

The application of the HMMs to the genomes of other nasopharyngeal commensals (*Haemophilus influenzae*, *Neisseria meningitidis *and *Staphylococcus aureus*) failed to identify any cases where the repeats had been horizontally transferred. A similar investigation of all publicly available complete streptococcal genomes, encompassing twelve species other than *S. pneumoniae*, also detected few instances of these repeat elements (Additional file 1). The sole representative genome of the most closely related species to *S. pneumoniae*, *S. mitis *B6 [24], contained 104 BOX elements (a mean density of 0.048 kb^-1^), slightly lower than the mean of 122 in the pneumococcal chromosomes (a mean density of 0.057 kb^-1^). By contrast, the density of SPRITE sequences in *S. mitis *is about half that of the pneumococcus, and there are only 9 detected instances of RUP in *S. mitis *B6. As *S. mitis *and *S. pneumoniae *are able to exchange DNA, it is not clear whether the repeats were present in their last common ancestor, or whether they have been acquired after speciation and subsequently spread horizontally. By contrast, all three repeat types are almost entirely absent from the genome of *S. sanguinis*, the only other mitis group streptococci to have been sequenced. Hence the most parsimonious conclusion is that these elements have spread in the pneumococcal chromosome subsequent to the divergence of the more distantly related members of the mitis group.

The only other streptococcal species to have a comparatively high number of detected repeats was *S. suis*, all genomes of which had 11 boxC elements. These were found to coincide with previously discovered repeats, annotated as 'RepSU1', on the complementary strand of the genome in strains SC84, P1/7 and BM407 [25]. Further analysis revealed the presence of two novel families of BOX-type elements in these genomes, composed of a total of seven different subsequences in particular permutations. One is bounded by boxA and C modules, both of which are around 50 nt long, as are the pneumococcal equivalents. The RepSU1 elements accounted for only the smallest BOX-type repeats of this type, equivalent to A_1_C_1 _BOX sequences. The other family has a boxE sequence at the 5' end and a boxF module at the 3' end; these motifs are comparatively large, having mean sizes of 115 nt and 133 nt respectively. Both types are found surrounding the same type of intervening boxB modules; however, the boxAC-flanked elements are also sometimes found having boxD modules, always in addition to boxB modules. Hence the diversity of *S. suis *BOX elements appears to be greater than that of the *S. pneumoniae *equivalents.

### Disruption and Modification of Genes Resulting from Repeat Element Insertion

BOX, RUP and SPRITE elements are frequently found together in clusters, and appear to have inserted into one another on a number of occasions. These spatial groupings may reflect a common preference for insertion sites, or a general tolerance of insertions in certain regions of the chromosome. However, repeats are also found interspersed within pseudogenes and regulatory sequences. It is known that BOX insertions can affect the expression of nearby genes [16,17]; another example where they might impact on the transcription of an operon is upstream of the *trp *gene cluster. In many Gram positive species, this operon is regulated by two copies of the T box riboswitch, which binds uncharged tRNA. Whilst streptococci have previously been thought to only have a single copy [26], in fact the pneumococcus has two, separated by a A_1_B_2_C_1 _BOX element. This results in the formation of a compound 5' untranslated region nearly a kilobase long, composed of three elements that, given their individually tightly folded structures, seem likely to fold largely independently.

A number of protein coding genes are disrupted by repeat insertions. Instances found in genome annotations include orthologues of the *S. pneumoniae *TIGR4 CDS SP_0243, encoding the extracellular binding protein for a putative iron ABC transporter, which is disrupted by the insertion of a RUP element in all the other pneumococcal genomes except *S. pneumoniae *AP200, 670-6B and TIGR4 itself. However, another CDS encoding part of the same ABC transporter (SP_0241 in TIGR4) is disrupted through frameshift mutations in these three strains. Both of these CDSs appear to be intact in several incompletely sequenced *S. mitis *strains, which lack the alternative *pit2 *iron transport system found on Pneumococcal Pathogenicity Island 1 [27]. SPN23F05190 (TIGR4 orthologues SP_0574 and SP_0575), encoding a restriction endonuclease in *S. pneumoniae *ATCC 70069, has a RUP insertion in *S. pneumoniae *TIGR4 and D39, whilst the orthologous gene in *S. pneumoniae *AP200 has been disrupted through the insertion of an IS element. Further examination of the repeat insertions reveals a RUP insertion that has knocked out a serine/threonine protein kinase, previously annotated as two separate CDSs (e.g. SPN23F18490 and SPN23F18500 in *S. pneumoniae *ATCC 700669; SP_1831 and SP_1832 in *S. pneumoniae *TIGR4), in all strains except *S. pneumoniae *Taiwan 19F-14 and TCH8431/19A. BOX elements can also cause gene disruption through insertion: a gene encoding a DNA alkylation repair protein is disrupted by a BOX insertion in all the available pneumococcal sequences, whilst an E_1_B_1_F_1 _element appears to have inserted into an acetyltransferase pseudogene in the sequenced *S. suis *genomes. Hence the mobility of these repeats has the potential to contribute to phenotypic polymorphism in the *S. pneumoniae *and *S. suis *populations.

### The Formation of Expressed Open Reading Frames by Large BOX Elements

Fifty-eight CDSs in the *S. pneumoniae *ATCC 700669 annotation overlap with BOX elements. In 36 cases, this corresponds to the extreme 3' end of a gene, with the BOX repeat encoding the stop codon; in some cases, these correspond to well-characterised genes such as *folE*, *mtlD*, *dnaJ *and *glgP*. However, alignments with non-pneumococcal orthologues do not provide strong evidence for truncation of the encoded polypeptide in any case, especially when the relatively weak conservation of the extreme C terminal portion of proteins is taken into account.

A further 19 sequences, which appear to encode proteins on the basis of GC frameplot and correlation scores [28], with little or no functional annotation were found to be mostly, or wholly, encoded by BOX elements. Pneumococcal BOX repeats can extend to over 500 bp in length, and these larger elements tend to encode an open reading frame on both strands. Of the CDSs encoded mainly by BOX sequence, all but two (SPN23F00880 and SPN23F08320) were annotated on the opposite strand of genome to that on which the BOX elements are marked. None of the translated BOX-encoded CDSs exhibited significant similarity with any sequence in the public databases other than matches to hypothetical proteins annotated in mitis group streptococcal genomes.

**Figure 6:**
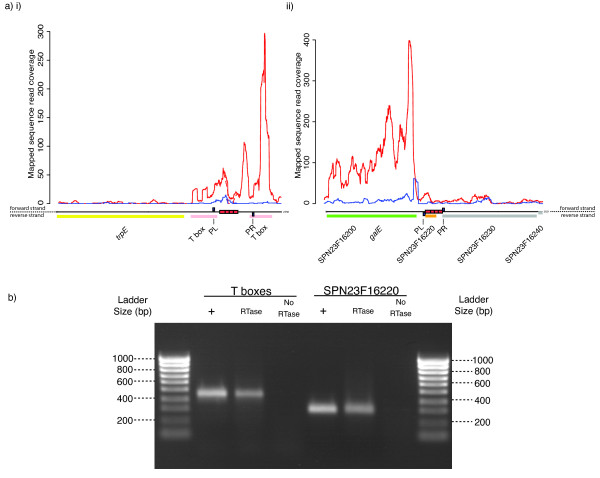
**Repeat sequence expression congruent with genome annotation**. a) All RNA-seq data is shown as plots of read coverage against the annotation of the represented genomic locus. Along the bottom of these panels, CDSs and non-coding RNAs, coloured according to function (see ref. [20]), are represented as blocks above or below the scale line, depending on their orientation. BOX repeats are shown as red blocks on the scale line. Primer binding sites are indicated by blue blocks labelled using dashed lines. Above the annotation, as part of the coverage plots, blue lines indicate transcription of the upper strand of the genome, while red lines show transcription of the reverse strand. Solid lines represent the result of fully redundant mapping, where reads mapping to multiple sites on the chromosome are randomly distributed between them. Dashed lines represent locally redundant mapping, where reads that might map to regions outside the displayed locus are excluded from the graph (see Methods). i) The region upstream of the *trp *operon. The *trpE *gene is adjacent to two T box riboswitch motifs separated by an intervening BOX element, represented as four adjacent red boxes representing the A_1_B_2_C_1 _structure of the repeat. The RNA-seq data suggests the T box motifs and BOX element are cotranscribed as a composite element, repressing the transcription of the downstream biosynthetic operon. ii) Locus surrounding SPN23F16220. This small CDS is annotated as being encompassed by a BOX element. RNA-seq data suggested it was cotranscribed with SPN23F16230, present on the other side of the repeat relative to the more highly expressed *galE *gene. b) RT-PCR to confirm transcription of these BOX elements. The positions of the primers used in these reactions are indicated by the blue boxes labelled PL (left primer) and PR (right primer) in a) i) and ii). In each case, the three lanes correspond to a positive control reaction using a genomic DNA (+), a test using cDNA produced through reverse transcription of an RNA sample (RTase) and a negative control using a non-reverse transcribed RNA sample (No RTase). The bands indicate that these BOX elements are expressed, as suggested by the RNA-seq data.

In order to determine whether these genes are expressed, we used directional RNA sequencing data [29], which allows transcription to be studied at very high resolution even in repetitive regions of the chromosome [30]. In the case of SPN23F16220 (Figure 6a, ii), the transcription follows the direction expected from the annotation, with the BOX element forming a 3' extension to the upstream three CDS operon, as confirmed by RT-PCR (Figure 6b). Entirely encompassed within this PCR product is a 42 aa predicted protein encoded by an A_1_B_2_C_1 _BOX. Also confirmed to conform to the genome annotation is the BOX element lying between the T box motifs upstream of the *trp *operon (Figure 6a, i). The pneumococcal culture from which the RNA was extracted was grown in nutrient-rich conditions, hence the T box motifs are expressed, but the downstream *trp *operon is not. Therefore it appears that the riboswitches are still able to function as a regulatory structure, despite the intervening BOX element. Hence, as anticipated from the genome sequence, BOX elements can be transcribed as extensions to both the 5' and 3' regions of operons.

**Figure 7:**
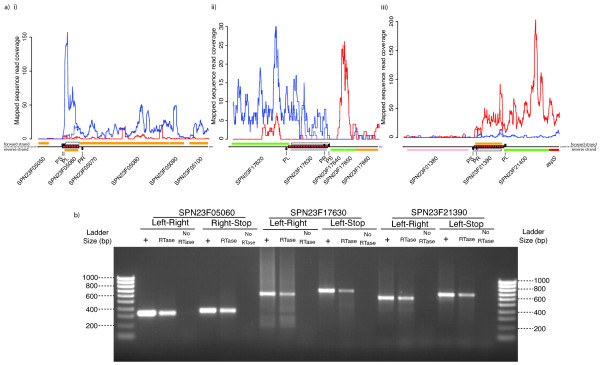
**Potential misannotation of the *S. pneumoniae *ATCC 700669 genome**. RNA-seq data is displayed as described in Figure 6. In addition to the published annotation of *S. pneumoniae *ATCC 700669, dashed boxes indicate alternative open reading frames encoded by BOX elements. a) i) The locus around SPN23F05060, encoded by a BOX element. The CDS is annotated on the bottom strand, but the RNA-seq results indicate it is co-transcribed with the operon on the top strand. ii) This BOX element appears to be cotranscribed with the upstream SPN23F17620 CDS at a low level, rather than encoding the 603 bp putative CDS SPN23F17630. iii) The BOX element encompassing putative CDS SPN23F21390 appears to transcribed on the reverse strand, along with the neighbouring CDSs. b) For each of the three loci displayed in a), two experiments were performed, each as described in Figure 6. One, using PL (left primer) and PR (right primer) tested for expression of the BOX element itself. The second used PL or PR and PS (stop codon primer), which tested whether the full length open reading frame on the transcribed strand was expressed. At all three loci, both reactions were positive using a cDNA sample as template.

However, in three cases, (SPN23F005060, SPN23F17630 and SPN23F21390), the direction of transcription indicated by the RNA-seq data contradicted the predicted CDS, appearing instead to be continuing from the adjacent operon (Figure 7a). SPN23F005060 is contained within a small 289 bp repeat likely to form a 5' extension to the downstream operon. The relatively high density of reads mapping to this BOX element may reflect mismapping of sequences that correspond to a different, more highly expressed repeat (as the level of locally redundant mapping is lower, and hence more congruent with the level of transcription of the rest of the operon), or indicate that the repeat functions as a transcriptional attenuator due to its highly folded structure. The BOX-encoded putative CDSs SPN23F17630 and SPN23F21390 form long (649 bp and 604 bp, respectively) 3' structures. The cotranscription of these elements in the direction indicated by the RNA-seq data was confirmed by RT-PCR in all three examples (Figure 7b), implying the annotation is likely to be erroneous.

However, in all three cases, there is also an ORF in the transcribed direction; rather than the start codon being in boxC and boxA encoding the stop codon, as predicted, boxC instead encodes the start codon and the stop codon lies beyond the BOX element. These expressed, BOX-encoded potential CDSs are indicated as dashed boxes in Figure 7a. Further RT-PCR confirmed that the RNA extended not just to the end of these BOX elements, but extended as far as the stop codon of these ORFs (Figure 7b). However, the proteins encoded by these ORFs also failed to significantly match any sequences other than hypothetical CDSs from mitis group streptococci and lacked good candidate Shine-Dalgarno sequences. Nevertheless, this confirmed that these 5' and 3' operon adducts, formed by BOX elements, have the potential to become nascent protein coding sequences.

## Discussion

The three families of small interspersed repeats found in the pneumococcal chromosome are found, albeit at a reduced frequency, in the closely related species, *S. mitis*, and very infrequently in other streptococci. These include the previously unidentified SPRITE repeat, which resembles a Rho-independent terminator element in its secondary structure. This is quite unlike the structures of the BOX and RUP elements, which are much more tightly folded and include their TIR hybridised to one another as parts of duplexes. A likely consequence of this form is the observed strong enrichment of this element close to the 3' ends of convergently transcribed CDSs, such that it does not disrupt normal gene expression patterns.

Even the naturally transformable oral streptococcus *S. sanguinis*, also part of the mitis group, lacks these elements. This implies that the repeats are unlikely to fulfil any of the possible important functions that might be ascribed to repeated sequences: for instance, chromosome packaging, aiding with replication or incorporation of horizontally transferred DNA. Furthermore, their distribution within the *S. pneumoniae *ATCC 700669 chromosome, resembling as it does the pattern of IS elements in being enriched between convergently transcribed CDSs, is suggestive of the main alternative explanation of their prevalence: that they are parasitic, non-autonomously mobile elements.

Based on their distribution between different streptococci, it appears that the repeats are likely to have been acquired subsequent to the divergence of the mitis group species. Two possible hypotheses may be advanced to explain the current distribution of repeats in the pneumococcus; one is that they may have been present in the last common ancestor of *S. pneumoniae*, and the position of some repeat insertions in this progenitor subsequently conserved amongst all pneumococcal strains. Alternatively, the repeats may have been acquired by *S. pneumoniae *and then spread horizontally through the population, resulting in the repeats being fixed at certain chromosomal loci over time. This second scenario is likely to be more sensitive to negative selection against the repeat insertions. In either case, a period of relatively rapid spread seems to have occurred in the population's past, which now seems to have abated. The proportion of repeats that are 'core' is similar to the proportion of 'core' CDSs in the pneumococcal pan-genome [31], and there are few insertions unique to any given chromosome that would indicate recent transposition events, contrasting with the distribution of IS elements between chromosomes.

The only other sequenced streptococcal species to have acquired BOX-type repeats is *S. suis*, which is also able to colonise the human nasopharynx, suggesting there may be a common source of these sets of elements. Although the *S. suis *BOX elements are present at a lower density in the chromosome, they are more diverse. It is difficult to assess how 'active' these elements are in this species, given the closely related nature of the currently sequenced *S. suis *genomes [25,32], but in the current sample there is little evidence that they are more mobile than in *S. pneumoniae*. Hence in both species, these elements appear to be currently dormant.

One reason to suggest there may be selection against any mechanism that mobilises such elements is the disruption of CDSs by repeat insertion, which is evident in both *S. pneumoniae *and *S. suis*. However, there is also the potential for the formation of novel ORFs by BOX elements. Again, this is observed in both species; as well as the pneumococcal instances, there are two CDSs in the *S. suis *genomes that appear to be intact despite containing box modules (SSUSC84_0055 and 0899 in *S. suis *SC84) and three that are mostly, or entirely, encoded by BOX elements (SSUSC84_0048, 0112 and 0453 in *S. suis *SC84). The RNA-seq and RT-PCR data suggest that in some cases in *S. pneumoniae *such elements are transcribed, and have the potential to become nascent CDSs. Such instances appear to represent the consequences of three proposed properties of BOX elements: firstly, their mobility allowing them to insert into transcribed regions of the genome; secondly, the formation of an open reading frame on both strands of the element, and thirdly, their modular nature allowing them to expand to longer forms.

Whether the polypeptides they encode are actually expressed is not clear; it seems more likely that they are transcribed as untranslated regions. If so, they may influence the levels of expression of co-transcribed genes; those elements forming 3' adducts to operons are likely to form stem-loop structures that may impede the action of 3'→5' exonucleases, the primary RNA degradation pathway in bacteria, thereby stabilising the transcript. However, ERICs are capable of triggering endoribonucleolytic cleavage of transcripts, depending on the orientation of the element and the sequence of the operon, and CE can also trigger cleavage of mRNA. Hence the overall impact of a repeat insertion into an operon is difficult to predict, and is liable to change with the variation in the length of the BOX element and the context of the insertion site. Unfortunately, the sequence read coverage across operons with current RNA-seq techniques is too inconsistent to make any firm inferences about the impact of these BOX elements [30].

The simplest mechanism by which these repeats may affect transcription is through acting as terminators, especially given the resemblance of SPRITE sequences to such structures. Such a function has been previously been proposed to be performed by a BOX element [17]. There is also a precedent for repeats having a similar impact in another nasopharyngeal commensal and pathogen: the 10 bp DNA uptake sequences (DUS) of *N. meningitidis *which, when found in close proximity to one another, tend to be inversely orientated, allowing them to form a stem loop structure predicted to act as a terminator [33]. *S. pneumoniae*, although naturally transformable, is not known to have any DUS as *N. meningitidis *and *H. influenzae *do, and partial SPRITE sequences were not sufficiently abundant to suggest the element described here is a composite of DUS pairs. It seems likely, in fact, that the prevalence of the repeat families present in the pneumococcal chromosome exemplifies a potential disadvantage of the intrinsically competent lifestyle these three respiratory pathogens have adopted: the risk of acquiring genomic parasites that may cause considerable disruption whilst they remain mobile.

## Conclusions

There are three families of small interspersed repeats in the *S. pneumoniae *chromosome: BOX, RUP and SPRITE. BOX-type repeats are also prevalent in the *S. suis *chromosome. The pneumococcal repeats appear to be parasitic, non-autonomous mobile elements that seem to have spread mainly during a burst of transposition subsequent to the divergence of *S. pneumoniae *and *S. mitis *from the other mitis group streptococci. BOX elements vary in size significantly, and are found to form expressed open reading frames that may constitute potential novel protein coding sequences or untranslated adducts to pre-existing operons.

## Methods

### Identification and Annotation of Repeat Sequences

The sequence of *S. pneumoniae *ATCC 700669 [20] [EMBL accession code: FM211187] was searched for repeats longer than 50 bp using RepeatScout [34]. For each of the three families identified, multiple sequence alignments were produced with MUSCLE [35], which were used to generate Hidden Markov Models (HMM) using HMMER1.8 (more recent versions of HMMER have not been optimised for searching long nucleic acid sequences for short motifs) [36]. In order to define the modular nature of BOX elements, HMMs representing boxA, B and C sequences individually were produced using available sequence data [14,37]. Sequences identified with these initial models were then aligned and used to produce the final HMMs used in this study; cutoff score thresholds were determined empirically from the distribution of scores for all hits throughout the genome. A composite BOX element was defined as two or more adjacent boxA, B or C modules. The same approach was used to generate HMMs for the repeats identified in *S. suis*. Thorough *de novo *searches for novel interspersed repeats in other species were not conducted.

In a number of cases where annotated repeat sequences overlapped, it was evident that one element had inserted into another. In such cases, for each repeat in the pair, a realignment of one repeat with the appropriate HMM was attempted using the concatenated flanking sequences of the other repeat, effectively excluding the sequence of the other element. If one of the elements had a greater bitscore when realigned in such a manner, it was reannotated as a split feature into which the other repeat had inserted.

HMM logos were produced using LogoMat-M [38]. Secondary structure predictions were produced from a multiple alignment of 30 repeat sequence examples (a random sample in the base of RUP elements; only BOX elements with the canonical A_1_B_1_C_1 _structure were used) using RNAalifold [39]. The HMMs for the *S. pneumoniae *and *S. suis *repeats, and a program to automate their annotation for viewing in Artemis [40], are made freely available from ftp://ftp.sanger.ac.uk/pub/pathogens/strep_repeats/. The annotation of repeat elements in complete *S. pneumoniae*, *S, mitis *and *S. suis *genomes is also available from this site. A revised annotation of *S. pneumoniae *ATCC 700669 has been submitted to the EMBL database.

### Definition of Orthologous Repeat Sequences

Of the 14 available complete pneumococcal genomes in the EMBL database (Additional file 1), all except *S. pneumoniae *R6 (a laboratory derivative of *S. pneumoniae *D39, the sequence of which is also available in the database) were analysed. For each annotated repeat element, 250 bp of upstream and downstream flanking sequence were concatenated into a single 500 bp string. All pairwise sequence comparisons between strings corresponding to repeats of the same type were performed using BLASTN [41]. The alignments with an E value smaller than 10^-25 ^were then used to cluster the strings into groups, corresponding to orthologous repeat insertions, using OrthoMCL [42]. The inflationary parameter used in clustering was set to 3, the smallest integral value that did not cluster a pair of insertions within the same genome together (i.e. identify 'paralogous' insertions). The IS elements in Figures 3 and 4 correspond to all the annotated IS element transposase CDSs in the *S. pneumoniae *ATCC 700669 genome; however, in order to identify orthologous IS elements in different pneumococcal genomes, a consistent annotation across the genomes was required. To automate this, such repeats were identified as BLASTN matches to defined elements in the IS database [43] with a nucleotide identity >95% and a length >90% of that of the reference sequence. Insertions of the same IS element type were then clustered as described for the small interspersed repeats, and the results for all IS elements subsequently combined to generate the data used in the graph.

### RNA Sequencing and RT-PCR

RNA sample extraction and processing, and cDNA sequencing, were carried out as described in Croucher *et al *[29]. The template for the RT-PCR reaction was generated from an independent RNA sample extracted from cultures of *S. pneumoniae *ATCC 700669 at a density of OD_600 _= 0.8 growing statically in Brain Heart Infusion broth (Oxoid) at 37°C. RT-PCR used the primers detailed in table 2 in a touchdown reaction (denaturing step of 95°C for 30 s; annealing temperature, held for 30 s, reduced from 60°C to 55°C over 5 cycles, then maintained at 55°C for a further 25 cycles; extension conditions of 1 min at 72°C). The reaction consisted of 45 μl PCR Platinum Supermix (Invitrogen), 2 μl of a 10 μM solution of each primer (Sigma) and 1 μl template (either 10 ng μl^-1 ^genomic DNA, 500 ng μl^-1 ^RNA or the cDNA generated from the 500 ng μl^-1 ^RNA sample).

**Table 2:** Sequences of primers used in this study

Primer Name	Primer Sequence (5'-3')
05060_PL	TCAAACCACGTCAGCTTCAC
05060_PR	CTTGTCCACCACTTCTGCAA
05060_PS	TAAAATCATTTTATACTCTTCGAAAATCTC
16220_PL	ACCTGATAAAATTTCAGTAAAATGC
16220_PR	TGTAAACGAGTAAAAGCGAAT
17630_PL	GAAGCCAAAACCTTCATCCA
17630_PR	CAGGCTGCTCAAAACACG
17630_PS	TTCACCTTTTGTGGATTGGTC
21390_PL	TGTTCATATTCTAGGAAGATTTGTTGA
21390_PR	GCAGGTTGCTCAAAACACTG
21390_PS	TCCATAATATCTATAGTGGATTTACCC
Tbox_PL	AATCTGCAATCGCAGCTAGG
Tbox_PR	CGTTACCAACGCCCTCAC

### Sequence Read Mapping

Sequence reads were mapped as paired end data using BWA [44]. The orientation of the second read in correctly mapped pairs was reversed using Samtools [45] before producing coverage plots, in order to maintain the directional fidelity of the data. In order to generate the plots in Figures 6 and 7, the 'XA' note in the alignment file was used to identify alternative mapping locations. All reads were used to generated the fully redundant plot; reads with alternative mapping loci only within the displayed region were maintained in the set used to generate the 'locally redundant' plot, whilst those that mapped equally well to sequences outside of the displayed region were excluded. This allows reads that come from a specific BOX element, but cannot be unambiguously assigned to a particular boxB module therein, to be retained within the 'locally redundant' plot. The RNA-seq data has been submitted to the European Nucleotide Archive with accession code ERR015608.

## Authors' contributions

NJC, JP and SDB conceived the project; NJC performed the experiments; NJC and GSV analysed the data; NJC and SDB wrote the manuscript. All authors approved the final draft.

## Supplementary Material

Additional file 1 **This table shows the number of *S. pneumoniae *BOX, RUP and SPRITE repeats, and the number of *S. suis *BOX repeats, found in each of the publicly available complete streptococcal genome sequences**. Click here for file
