# Supplementary material for: Identification, variation and transcription of pneumococcal repeat sequences
Source: BMC Genomics. 2011 Feb 18;12:120. doi: 10.1186/1471-2164-12-120 (PMC3049150; doi:10.1186/1471-2164-12-120)
Supplement: Additional file 1 — This table shows the number of S. pneumoniae BOX, RUP and SPRITE repeats, and the number of S. suis BOX repeats, found in each of the publicly available complete streptococcal genome sequences. [file 1471-2164-12-120-S1.DOC]

|  | ***S. pneumoniae* Repeats** | | | | | ***S. suis* Repeats** | | | | | |
| --- | --- | --- | --- | --- | --- | --- | --- | --- | --- | --- | --- |
| **Genome** | RUP | SPRITE | boxA | boxB | boxC | boxA | boxB | boxC | boxD | boxE | boxF |
| *Streptococcus agalactiae* 2603V/R | 0 | 0 | 0 | 0 | 0 | 0 | 0 | 0 | 0 | 0 | 0 |
| *Streptococcus agalactiae* A909 | 0 | 0 | 0 | 0 | 0 | 0 | 0 | 0 | 0 | 0 | 0 |
| *Streptococcus agalactiae* NEM316 | 0 | 0 | 0 | 0 | 0 | 0 | 0 | 0 | 0 | 0 | 0 |
| *Streptococcus dysgalactiae* subsp. equisimilis GGS_124 | 0 | 0 | 0 | 0 | 0 | 0 | 0 | 0 | 0 | 0 | 0 |
| *Streptococcus equi* subsp. equi 4047 | 0 | 0 | 0 | 0 | 0 | 0 | 0 | 0 | 0 | 0 | 0 |
| *Streptococcus equi* subsp. zooepidemicus H70 | 0 | 0 | 0 | 0 | 0 | 0 | 0 | 0 | 0 | 0 | 0 |
| *Streptococcus equi* subsp. zooepidemicus MGCS10565 | 0 | 0 | 0 | 0 | 0 | 0 | 0 | 0 | 0 | 0 | 0 |
| *Streptococcus gallolyticus* UCN34 | 0 | 0 | 0 | 0 | 0 | 1 | 1 | 2 | 0 | 0 | 0 |
| *Streptococcus gordonii* str. Challis substr. CH1 | 0 | 1 | 1 | 0 | 1 | 0 | 0 | 1 | 0 | 0 | 0 |
| *Streptococcus mitis* B6 | 9 | 15 | 104 | 103 | 103 | 0 | 0 | 94 | 0 | 0 | 0 |
| *Streptococcus mutans* NN2025 | 0 | 0 | 1 | 1 | 2 | 0 | 0 | 4 | 0 | 0 | 0 |
| *Streptococcus mutans* UA159 | 0 | 0 | 1 | 1 | 2 | 0 | 0 | 3 | 0 | 0 | 0 |
| *Streptococcus pneumoniae* 670-6B | 105 | 28 | 123 | 200 | 121 | 0 | 0 | 94 | 0 | 0 | 0 |
| *Streptococcus pneumoniae* 70585 | 111 | 31 | 124 | 196 | 121 | 0 | 0 | 97 | 0 | 0 | 0 |
| *Streptococcus pneumoniae* AP200 | 101 | 26 | 119 | 163 | 117 | 0 | 0 | 89 | 0 | 0 | 0 |
| *Streptococcus pneumoniae* ATCC 700669 | 110 | 30 | 127 | 183 | 122 | 0 | 0 | 93 | 0 | 0 | 0 |
| *Streptococcus pneumoniae* CGSP14 | 103 | 29 | 128 | 195 | 122 | 0 | 0 | 93 | 0 | 0 | 0 |
| *Streptococcus pneumoniae* D39 | 106 | 28 | 117 | 160 | 110 | 0 | 0 | 85 | 0 | 0 | 0 |
| *Streptococcus pneumoniae* G54 | 102 | 29 | 119 | 170 | 116 | 0 | 0 | 92 | 0 | 0 | 0 |
| *Streptococcus pneumoniae* Hungary19A-6 | 105 | 30 | 125 | 187 | 121 | 0 | 0 | 95 | 0 | 0 | 0 |
| *Streptococcus pneumoniae* JJA | 102 | 30 | 126 | 189 | 124 | 0 | 0 | 95 | 0 | 0 | 0 |
| *Streptococcus pneumoniae* P1031 | 109 | 28 | 124 | 186 | 120 | 0 | 0 | 97 | 0 | 0 | 0 |
| *Streptococcus pneumoniae* R6 | 106 | 28 | 117 | 160 | 110 | 0 | 0 | 85 | 0 | 0 | 0 |
| *Streptococcus pneumoniae* Taiwan19F-14 | 101 | 29 | 118 | 168 | 116 | 0 | 0 | 89 | 0 | 0 | 0 |
| *Streptococcus pneumoniae* TCH8431/19A | 100 | 28 | 119 | 172 | 117 | 0 | 0 | 88 | 0 | 0 | 0 |
| *Streptococcus pneumoniae* TIGR4 | 108 | 25 | 128 | 195 | 127 | 0 | 0 | 97 | 0 | 0 | 0 |
| *Streptococcus pyogenes* M1 | 0 | 0 | 0 | 0 | 0 | 0 | 0 | 0 | 0 | 0 | 0 |
| *Streptococcus pyogenes* Manfredo | 0 | 0 | 0 | 0 | 0 | 0 | 0 | 0 | 0 | 0 | 0 |
| *Streptococcus pyogenes* MGAS10270 | 0 | 0 | 0 | 0 | 0 | 0 | 0 | 0 | 0 | 0 | 0 |
| *Streptococcus pyogenes* MGAS10394 | 0 | 0 | 0 | 0 | 0 | 0 | 0 | 0 | 0 | 0 | 0 |
| *Streptococcus pyogenes* MGAS10750 | 0 | 0 | 0 | 0 | 0 | 0 | 0 | 0 | 0 | 0 | 0 |
| *Streptococcus pyogenes* MGAS2096 | 0 | 0 | 0 | 0 | 0 | 0 | 0 | 0 | 0 | 0 | 0 |
| *Streptococcus pyogenes* MGAS315 | 0 | 0 | 0 | 0 | 0 | 0 | 0 | 0 | 0 | 0 | 0 |
| *Streptococcus pyogenes* MGAS5005 | 0 | 0 | 0 | 0 | 0 | 0 | 0 | 0 | 0 | 0 | 0 |
| *Streptococcus pyogenes* MGAS6180 | 0 | 0 | 0 | 0 | 0 | 0 | 0 | 0 | 0 | 0 | 0 |
| *Streptococcus pyogenes* MGAS8232 | 0 | 0 | 0 | 0 | 0 | 0 | 0 | 0 | 0 | 0 | 0 |
| *Streptococcus pyogenes* MGAS9429 | 0 | 0 | 0 | 0 | 0 | 0 | 0 | 0 | 0 | 0 | 0 |
| *Streptococcus pyogenes* NZ131 | 0 | 0 | 0 | 0 | 0 | 0 | 0 | 0 | 0 | 0 | 0 |
| *Streptococcus pyogenes* SSI-1 | 0 | 0 | 0 | 0 | 0 | 0 | 0 | 0 | 0 | 0 | 0 |
| *Streptococcus sanguinis* SK36 | 0 | 1 | 0 | 1 | 1 | 0 | 0 | 0 | 0 | 0 | 0 |
| *Streptococcus suis* 05ZYH33 | 0 | 0 | 1 | 0 | 11 | 46 | 43 | 49 | 15 | 33 | 17 |
| *Streptococcus suis* 98HAH33 | 0 | 0 | 1 | 0 | 11 | 47 | 44 | 49 | 15 | 34 | 17 |
| *Streptococcus suis* BM407 | 0 | 0 | 1 | 0 | 11 | 47 | 43 | 49 | 15 | 34 | 16 |
| *Streptococcus suis* GZ1 | 0 | 0 | 1 | 0 | 11 | 46 | 44 | 49 | 14 | 33 | 17 |
| *Streptococcus suis* P1/7 | 0 | 0 | 1 | 0 | 11 | 47 | 44 | 49 | 15 | 34 | 17 |
| *Streptococcus suis* SC84 | 0 | 0 | 1 | 0 | 11 | 47 | 44 | 49 | 15 | 34 | 17 |
| *Streptococcus thermophilus* CNRZ1066 | 0 | 0 | 0 | 0 | 0 | 0 | 0 | 0 | 0 | 0 | 0 |
| *Streptococcus thermophilus* LMD-9 | 0 | 0 | 0 | 0 | 0 | 0 | 0 | 0 | 0 | 0 | 0 |
| *Streptococcus thermophilus* LMG 18311 | 0 | 0 | 0 | 0 | 0 | 0 | 0 | 0 | 0 | 0 | 0 |
| *Streptococcus uberis* 0140J | 0 | 0 | 0 | 0 | 0 | 0 | 0 | 0 | 0 | 0 | 0 |
